# Dedifferentiated Liposarcoma of the Spermatic Cord: A Case Report and Review of the Literature

**DOI:** 10.7759/cureus.80264

**Published:** 2025-03-08

**Authors:** Yuki Matsui, Wahei Yanagida, Hirotaka Kishi, Jun Morita, Takashi Fukagai

**Affiliations:** 1 Department of Urology, Showa University School of Medicine, Tokyo, JPN

**Keywords:** chemotherapy, dedifferentiated liposarcoma, genitourinary oncology, histopathology, prognosis, radiotherapy, retroperitoneal sarcoma, soft tissue sarcoma, spermatic cord, surgical oncology

## Abstract

Spermatic cord liposarcomas are rare malignant neoplasms with no established level-one evidence-based treatment guidelines. Well-differentiated liposarcomas (WDL) lack metastatic potential but can transform into dedifferentiated liposarcomas (DDL), which are more aggressive. Due to the rarity of these tumors, treatment recommendations are primarily based on retrospective studies and extrapolated data from extremity sarcomas.

We report a case of a DDL managed with radical orchiectomy and en bloc resection, along with a literature review on spermatic cord liposarcomas, focusing on imaging, histopathology, and treatment strategies. Imaging techniques, such as magnetic resonance imaging (MRI) and computed tomography (CT), aid in tumor characterization, while histopathological examination remains the gold standard for diagnosis. Surgical management prioritizes R0 resection, as incomplete excision is associated with high recurrence rates. The role of adjuvant therapy remains controversial, with limited evidence supporting its routine use. In our case, complete resection with negative margins was achieved, and clinical follow-up was chosen over adjuvant treatment.

Given the aggressive nature of high-grade liposarcomas and their tendency for local recurrence, wide surgical excision with negative margins remains the primary treatment. The role of adjuvant chemotherapy and radiotherapy is unclear and may be considered in high-risk cases. Further research is needed to establish standardized treatment protocols.

## Introduction

Sarcomas of the genitourinary region are rare, comprising about 2% of all soft tissue sarcomas [1,2]. Malignant spermatic cord tumors are particularly uncommon, with an annual incidence of 0.3 cases per million [3]. The majority of spermatic cord masses are benign lipomas, making up 70-80% of cases [4]. Among malignant tumors, liposarcomas account for approximately 3-7% and are often misdiagnosed as benign lesions, leading to delayed treatment [4]. Liposarcomas are classified into well-differentiated, myxoid, pleomorphic, round cell, and dedifferentiated subtypes [5]. While well-differentiated liposarcoma (WDL) is the most prevalent, dedifferentiated liposarcoma (DDL) is rarer and often presents as a high-grade tumor with more aggressive behavior [5]. Diagnosis relies on imaging modalities such as ultrasound, CT, and MRI, while histopathology and immunohistochemistry, particularly Murine double minute 2 (MDM2) amplification, confirm the diagnosis [6,7]. Surgical resection with negative margins remains the cornerstone of treatment, but optimal management strategies, including the role of adjuvant therapy, remain controversial due to the rarity of these tumors. We report a rare case of DDL of the spermatic cord, highlighting the importance of early recognition and appropriate surgical intervention.

## Case presentation

**Figure 1:**
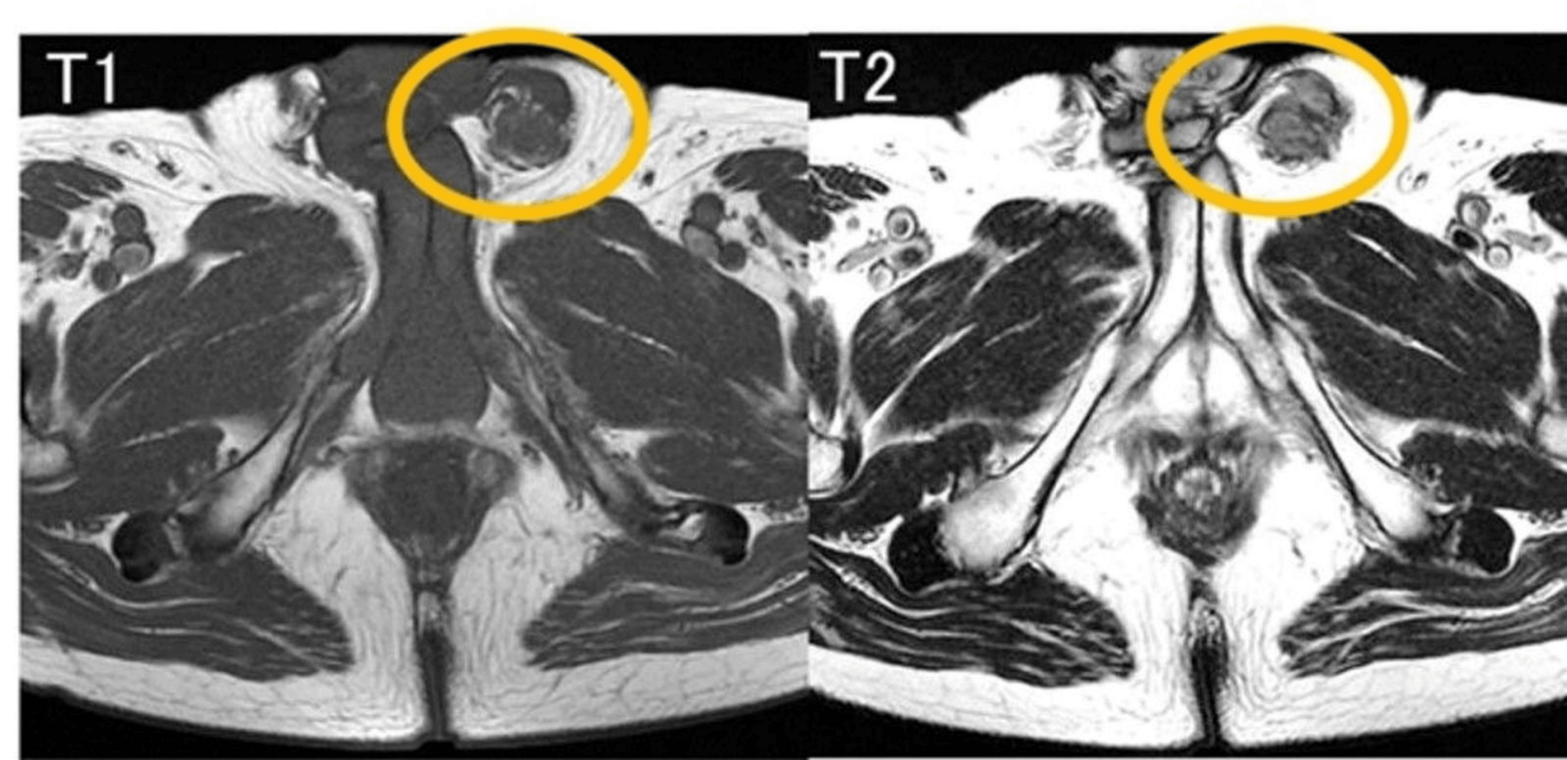
Preoperative imaging of dedifferentiated liposarcoma of the spermatic cord Both T1 and T2 and non-uniform high signal with diffusion weighting at the left spermatic cord site.

**Figure 2:**
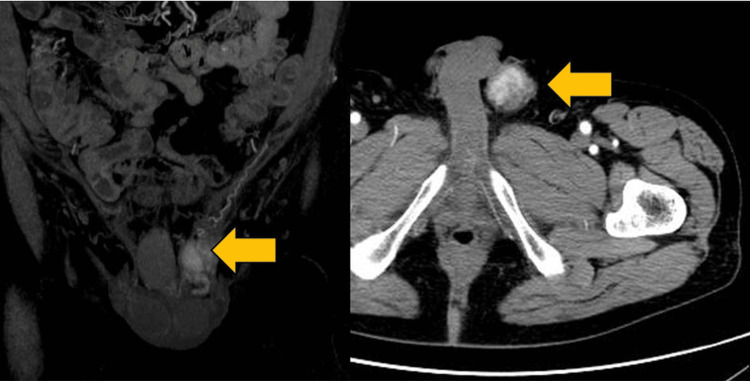
Coronal and axial enhanced CT images showing a solid tumor in the left spermatic cord (yellow arrows). Coronal (left) and axial (right) enhanced CT scans reveal a well-defined, solid mass (yellow arrows) in the left spermatic cord. The tumor appears hyperdense relative to the surrounding soft tissue structures.

**Figure 3:**
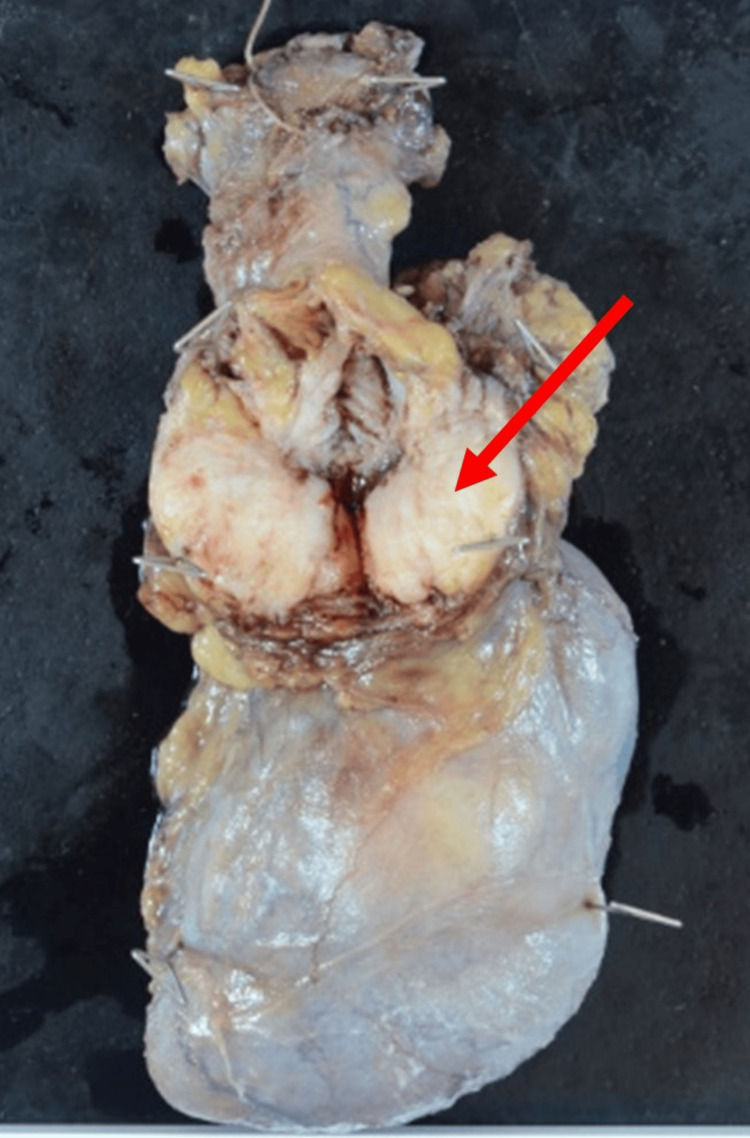
Surgical resection specimen of dedifferentiated liposarcoma The macroscopic cut surface of the specimen shows a white nodular mass, measuring 4 cm × 3.5 cm × 3 cm.

**Figure 4:**
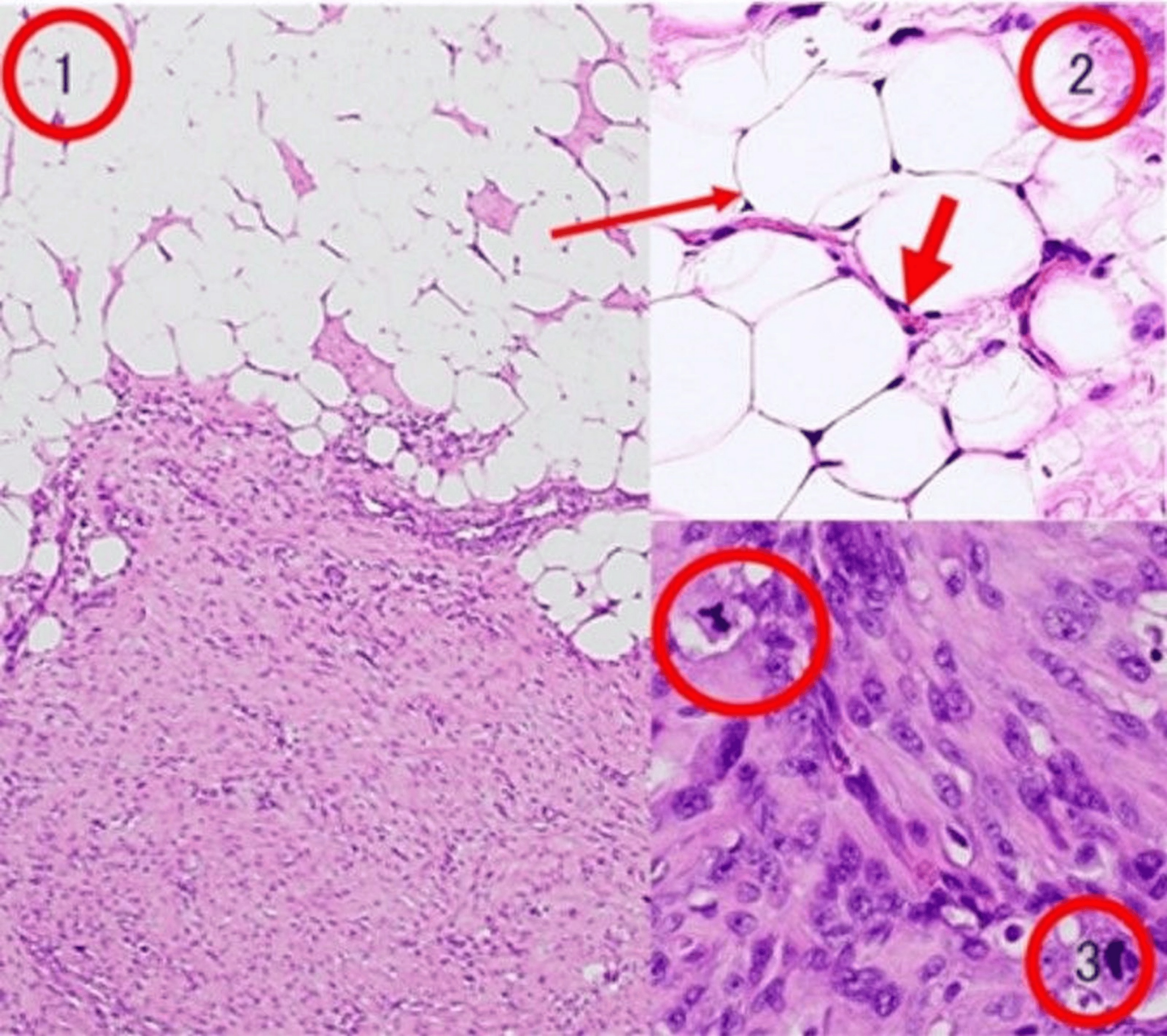
Histopathological findings of dedifferentiated liposarcoma 1: Well-differentiated liposarcoma with dedifferentiated components 2: Well-differentiated liposarcoma area shows that adipocytes are adjacent to cells with spindle-shaped nucleus. Anisocytosis of the nucleus is recognized, and fiber components are seen in the interstitium. 3: Dedifferentiated liposarcoma area is characterized by high cell density and strong nuclear deformity.

A 77-year-old man noticed a painless swelling in the left inguinal region. Physical examination showed a hard and immobile tumor mass about 4 cm in diameter size in the left inguinal region. A pelvic MRI showed a solid tumor with a low level of fatty-containing parts in the left spermatic cord (Figure 1). Enhanced CT of the chest and abdomen showed a solid tumor in the left spermatic cord (Figure 2). Regarding tumor markers, there was no elevation of CEA, CA19-9, AFP, HCG, or LDH in this case. No regional lymph node involvement or distant metastasis was identified during the workup. A left radical orchiectomy with wide local excision of the spermatic cord mass was performed en masse for both pathological diagnosis and curative treatment. The macroscopically cut surface of the specimen showed a white nodular mass measuring 4 cm × 3.5 cm × 3 cm (Figure 3). Histopathologic study of the resected specimen revealed a WDL with dedifferentiated components, negative resection margins, and normal testis. At the well-differentiated component site, adipocytes were adjacent to cells having a spindle-shaped nucleus, and the interstitium contained a fibrous component. At the poorly differentiated component site, cell density was high, the nuclear atypia was strong, and almost no fat component was observed (Figure 4). Immunostaining findings were positive for MDM2, CDK4, and P16. It was diagnosed as a dedifferentiated spermatic cord liposarcoma that had transitioned from the well-differentiated type. No adjuvant treatment was done, and 15 months of postoperative follow-up found no signs of recurrence and metastasis.

## Discussion

Soft tissue tumors account for approximately 1% of all tumors, with liposarcomas comprising 9.8-18% of soft tissue malignancies [8]. Among them, WDL is a locally aggressive neoplasm that lacks metastatic potential unless dedifferentiation occurs, at which point it transforms into DDL. DDL accounts for 4-5% of all liposarcomas and is often diagnosed only after significant tumor growth due to the absence of specific laboratory markers [8].

Imaging plays a crucial role in the identification and management of DDL. MRI typically reveals well-defined, lobulated lesions with heterogeneous signal intensity on T1- and T2-weighted images, reflecting the variable composition of fat, necrosis, and solid tumor components. Contrast-enhanced MRI and CT scans often demonstrate irregular enhancement patterns, with areas of necrosis and hypervascular solid regions suggestive of dedifferentiation. These imaging findings assist in differentiating DDL from benign lipomatous tumors and in determining the extent of local invasion and surgical resectability. Sonography has a high sensitivity (95-100%) for differentiating extratesticular from intratesticular lesions, with Doppler imaging showing paratesticular sarcomas as hypervascular tumors [9]. Given the aggressive nature of DDL, imaging not only aids in initial diagnosis but also plays a critical role in postoperative surveillance, where MRI and CT are utilized to detect early recurrence or distant metastases [10].

Histopathological examination remains the gold standard for diagnosis. The World Health Organization classifies liposarcomas into five subtypes: well-differentiated, dedifferentiated, myxoid, round cell, and pleomorphic [11]. WDL is further categorized into adipocytic, sclerosing, inflammatory, and spindle cell subtypes. DDL is histologically defined by the presence of non-lipogenic sarcomatous components, sometimes with admixed lipoblasts. Immunohistochemical markers, particularly MDM2 and CDK4, show nuclear expression in both spindle and adipocytic cells, aiding in the differentiation of liposarcomas from benign lipomas. Other markers, such as HMGA2 and p16, have also been implicated in the diagnostic process [6,7].

Despite literature accounts of surgeons’ experience with inguinal malignant neoplasms such as liposarcomas, standardized level-one evidence-based surgical management algorithms remain lacking. Most published data consist of case reports, retrospective analyses, and surgeon experience. Due to the rarity of spermatic cord liposarcomas, no single institution has accumulated enough cases to conduct randomized controlled trials. A Harvard Medical School study analyzing 362 patients estimated an annual incidence of 0.3 cases per million, which has remained stable over time [3].

Liposarcomas primarily exhibit local invasion rather than lymphatic spread [4]. High-grade subtypes are associated with higher recurrence rates and hematogenous dissemination to the bones and lungs. As a result, the widely accepted treatment approach includes en bloc resection of the mass, radical orchiectomy, and high ligation of the spermatic cord, with the primary objective of achieving negative surgical margins (R0 resection) [4]. While preoperative fine-needle aspiration (FNA) cytology has not been clearly established as a diagnostic tool, wide resection remains crucial due to the aggressive nature of these tumors, with a five-year mortality rate reported as high as 85% [6].

Surgical resection remains the cornerstone of treatment for spermatic cord DDL. The standard approach involves radical orchiectomy with en bloc resection of the tumor and high ligation of the spermatic cord. Given the high risk of local recurrence, achieving negative surgical margins (R0 resection) is crucial for improving long-term outcomes [4]. In cases where the tumor extends beyond the spermatic cord, wider resection involving adjacent structures such as the scrotal wall or inguinal canal may be necessary. Routine lymphadenectomy is generally not indicated, as DDL predominantly exhibits local invasion rather than lymphatic spread. However, in cases with radiologically evident lymphadenopathy or histopathological confirmation of nodal involvement, lymphadenectomy may be considered. Some reports suggest that lymph node involvement may be associated with more aggressive disease behavior, warranting closer follow-up [4]. Further studies are needed to establish clear guidelines regarding the role of lymphadenectomy in spermatic cord DDL. The extent of resection should be carefully planned based on preoperative imaging and intraoperative findings. If R0 resection is not feasible initially, re-excision may be considered to achieve complete tumor clearance. Given the potential for dedifferentiation and recurrence, a multidisciplinary approach involving surgical oncology, pathology, and radiology is essential in optimizing management strategies.

Currently, adjuvant chemotherapy and radiotherapy remain controversial, with no conclusive evidence supporting their routine use. A Memorial Sloan-Kettering Cancer Center study on 47 patients reported that 45% underwent adjuvant radiotherapy and 19% received chemotherapy, yet no significant therapeutic effect was demonstrated [12]. However, data from extremity sarcomas suggest that adjuvant radiotherapy may reduce local recurrence in high-risk cases with multiple recurrences, positive margins, and high-grade tumors [13]. Additionally, an Italian study reported a survival benefit for chemotherapy in high-grade extremity sarcomas, showing improved median overall survival from 46 months to 75 months [14]. In France, researchers found a significant decrease in local recurrence rates with radiotherapy, though no impact on overall survival was observed [15]. A recent Japanese case study described a dedifferentiated liposarcoma managed with additional wide resection after an initial positive margin, achieving disease-free status without adjuvant therapy [8].

Recent genetic studies have provided insights into the molecular characteristics of DDL. A 2019 study from Japan analyzed 115 cases of DDL using whole-exome sequencing (WES) and RNA sequencing (RNAseq) [16]. The study identified frequent copy number alterations in 83 genomic regions and 812 genes, with specific amplifications in 1p32.1, 1q24.3, 4p16.3, and Xq21.1, as well as deletions in 9q34.11, 12q24.33, 13q32.3, and Xp22.33. These genetic changes have been correlated with clinical prognosis, suggesting a potential role in risk stratification and targeted therapeutic approaches. Further research is needed to validate these findings and explore their clinical applications.

In our case, a complete resection with 5 cm negative margins was achieved, and given the lack of clear guidelines or randomized trials recommending adjuvant therapy, aggressive clinical follow-up was chosen instead. Long-term surveillance with physical examination and cross-sectional imaging remains crucial to monitor for recurrence. Current recommendations for high-grade soft tissue sarcomas suggest postoperative surveillance with MRI or CT every 3-6 months for the first two years, followed by annual imaging thereafter for up to five years, as most recurrences occur within this period. In addition, PET-CT may be considered in select cases with a high suspicion of recurrence. Close clinical monitoring for local symptoms, such as a palpable mass or pain, is essential to detect early recurrence, as delayed intervention can significantly impact prognosis.

## Conclusions

DDL of the spermatic cord is an extremely rare malignancy with aggressive potential. Radical surgery, including high orchiectomy and wide tumor resection, is essential to achieving long-term, disease-free survival. Given the higher malignancy and recurrence risk of DDL compared to WDL, careful and prolonged postoperative follow-up is crucial for early detection of recurrence.

This case report reinforces the importance of assessing recurrence risk and emphasizes the need for long-term clinical surveillance. As research advances in the areas of genetic analysis and novel therapeutic strategies, treatment approaches and prognostic evaluations are expected to become more precise. However, the limitations of case reports must be acknowledged, and further multi-center case accumulation and analysis are needed to refine clinical guidelines and therapeutic protocols.
